# Study on Electrical Pitting Prevention Device of a Rotating Shaft Using Automatic Control Potential Balancing

**DOI:** 10.3390/ma15134510

**Published:** 2022-06-27

**Authors:** Dong Won Son, Tuo Zhang, Geesoo Lee

**Affiliations:** 1 Department of Mechanical System Engineering, Tongmyong University, Busan 48520, Korea; sdwg@hanmail.net (D.W.S.); zhangtuo2020@naver.com (T.Z.); 2 Department of Automotive Engineering, Tongmyong University, Busan 48520, Korea

**Keywords:** bearing voltage, bearing current, electrical power, electrical pitting, electric machine, electrical corrosion protection device

## Abstract

A rotating body consisting of a rotating shaft and bearings inevitably generates voltage and current. The potential difference between the bearing and the shaft is the main cause of electrical corrosion, which causes motor failure, shortened bearing life, and many safety issues. To prevent corrosion, passive shaft-grounding devices use conductive materials and brushes; however, these devices cannot be completely grounded, so there is a difference in local potential, and brush friction generates a shaft current. The cumulative effect causes electrical corrosion; therefore, in this study, an electrical corrosion protection device for the rotating power supply shaft was developed. It detected current and potential difference and established a feedback system on the rotating shaft. It also energized the rotating shaft using an external power supply to eliminate the potential difference on the shaft and reduce electrical corrosion. The result was prolonged motor life and improved stability, operating efficiency, and operability of related equipment. In this study, a rotating-shaft test rig was set up, and a constant current was applied to simulate the potential difference and verify the performance of the anti-corrosion device. Gradually, the design scheme was optimized; the potential difference on the rotating shaft was accurately quantified; and the goal of controlling the potential difference within 2 mV was achieved. Finally, the electrical corrosion protection device was applied to the rotating shaft of a merchant ship, and the current and potential difference on the rotating shaft were monitored for 30 days. The results showed that the device had excellent performance in reducing the potential difference on the rotating shaft and preventing electrical corrosion.

## 1. Introduction

A rotating body consisting of a shaft and a bearing will inevitably generate electrical discharge in electrical machines [1,2]. The bearing current is mainly generated by the influence of electrostatic and electromagnetic fields [3]: the current related to the electrostatic field causes shaft voltage, and the current related to the electromagnetic field is expressed as circulating current. Because both acyclic and cyclic currents are closely related to the potential difference across the bearing, voltage is often used as an indicator of bearing failure [4,5]. Machine bearing currents can lead to grid current distortion [6,7], power loss [8], shortened bearing life [9], motor failure [10], and many other safety issues that increase machine maintenance and repair costs. Numerous methods are used for galvanic ally corroded surface inspection, such as visual inspection [11], shaft current damage signal detection [12,13,14], scanning electron microscope (SEM) observation [15], and microwave non-destructive testing (NDT) [16]. Research has shown that the most common form of damage from bearing currents is pitting or burns on the bearing raceways and rolling element surfaces [17]. Surface damage to bearings and journals takes on a linear, jagged characteristic shape and can also aggregate into point craters [11]. The intensity of this damage depends on the density of the flowing current and the duration of its operation [18,19,20,21,22,23]. The potential difference caused by the bearing current is the main cause of electrical corrosion in rotating machinery such as motors and generators [24,25], so the current should be reduced to ensure that the system functions efficiently [26].

As early as 1924, Alger et al. recognized that the drive generates shaft voltage and current [27]. Erdman et al. pointed out that variable-speed drives using pulse width modulation (PWM) voltage-source inverters exacerbate bearing currents, so more research is needed on them and on mitigation techniques [28]. Von Jouanne et al. elaborated on issues associated with long-cable-fed PWM drive systems [29]. Distributed long cables can cause overvoltage at the motor terminals causing bearing insulation failure and inducing bearing currents [30,31]. Han et al. proposed that the bearing voltage waveform depends to a large extent on inverter output, cable and ground impedance, and high-frequency and bearing parameters [32]. Chen et al. pointed out that the high-frequency common-mode voltage of the inverter and the stray capacitance of the motor are the key causes of bearing current generation [33]. Adabi et al. analyzed the influence of machine design on the stray capacitance factor, studied the relationship between the stray capacitance parameters of the bearing current model, and gave the relevant analytical formulas [34,35]. The schemes currently used to eliminate inverter-induced bearing currents mainly include shaft grounding systems [36,37], electrostatic shielding [38], and dual-bridge inverters [39]. However, the above solutions must be combined with machine design, which is very difficult to implement in the drive system. First, to ensure safety, many mechanical industries require lower potential differences [40]. For example, commercial engine companies such as MAN Diesel and BMW limit the potential difference between rotating shafts and bearings to 50 mV. At present, it is difficult for existing devices to keep within this limit. Second, in machines such as ships, even when the passive rotating-shaft-grounding device is in a normal state, there is a slight resistance between the shaft and the hull that cannot be completely grounded [41]. Moreover, the passive rotating-shaft-grounding device requires periodic maintenance of the slip-ring assembly and brushes because if performance degrades, localized electrical corrosion may occur. To solve these problems, a separate slip ring is being developed to monitor the potential between the shaft and ground in real time. If resistance between the shaft and the hull increases sharply, the separate slip ring and power supply rotate the shaft. However, at present, electric corrosion-prevention devices that use power supplies are rarely used in shipbuilding. Therefore, before using such a device, it is necessary to study the specifications and manufacturing methods [42]. The hull of the ship mainly uses the cathodic protection method, and the external sacrificial power-supply anode keeps the surface potential of the hull in a noncorrosive condition, also known as the external power-supply-type anti-corrosion device [43]. Han et al. proposed the use of insulating shafts to reduce the potential difference on the shaft. The research results show that the use of insulating shafts has a poor blocking effect on the induced current generated by the electromagnetic field, and the potential difference can only be reduced by 70% at most. [32]. Muetze et al. proposed the release of the voltage build-up on the bearing by electrostatic charge dissipation. The shaft current is released through the high-conductive fiber ring, which can only reduce the shaft voltage to 4–7 V and therefore does not meet the safety requirements of current equipment [37]. Bharatiraja et al. suppressed the bearing current by eliminating the common-mode voltage by introducing a switching algorithm for the fourth arm circuit (FAC). The experimental results show that this scheme has a good effect in eliminating common mode voltage and suppressing bearing current, but in the actual operation of the machine, the shaft current does not only include common mode current, so the effect of this scheme in suppressing bearing current still needs to be validated in actual device work [26]. Most previous studies had focused on a passive-shaft grounding device; there has been limited research on reducing the potential difference. The aim of this study was to design the active shaft grounding with the algorithm of the automatic control potential balance system within 2 mV, which was a new idea for designing the rotating shaft anti-corrosion device.

To solve the electrical corrosion problem, it was very important to supply and control the current to the rotating shaft precisely to balance the potential difference. An algorithm was used to detect the potential difference, calculate the corresponding balance current, and deliver it to the rotating shaft. In this study, a power supply was added to the shaft-grounding device, and PWM technology was used to drive the voltage source inverter (VSI). A complete feedback regulation system was established by designing a potential difference detector, power-control device, monitoring device, and grounding-related accessories. The electrical corrosion protection device of the power supply shaft measured the potential difference between the rotating body and the bearing and calculated the current corresponding to the potential difference flows to the rotating shaft. In this way, the potential difference on the rotating shaft was eliminated, and the various parts of the rotating body were prevented from being burned or damaged from electrolysis, thereby prolonging its service life. The experimental results showed that the anti-corrosion device controlled the potential difference on the rotating shaft within 2 mV, which was superior to the performance of the conventional shaft-grounding device, thus verifying its application to industry. 

## 2. Experimental Setup

### 2.1. Principle of Prevention of Electrical Pitting

**Figure 1:**
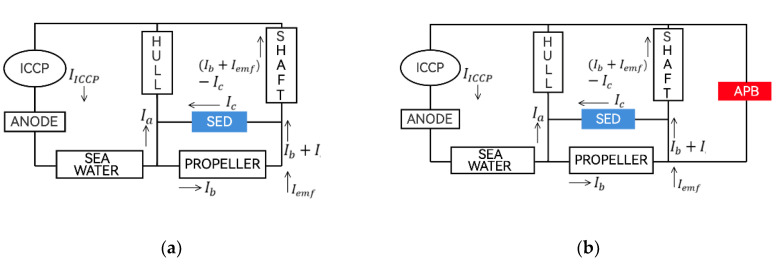
Circuit diagram of a marine electrical corrosion protection device: (**a**) passive shaft-grounding device and (**b**) electrical pitting prevention device with APB feedback adjustment system.

The circuit diagram of the existing marine passive shaft-grounding device and the electrical corrosion protection device of the power supply shaft is shown in Figure 1a. The traditional passive shaft-grounding device is affected by the current conduction of the hull and the propeller and cannot be completely grounded. As shown in Figure 1b, the design idea of this study is to add an automatic control potential balancing (APB) feedback adjustment system on the passive shaft-grounding device to balance the current and eliminate the potential difference on the rotating shaft.

According to Figure 1a, we can derive the conclusion:

(1) $$V_{shaft}=R_{shaft}\times \left\{\left(I_{b}+I_{emf}\right)-I_{c}\right\}$$

where $V_{shaft}$ is the shaft voltage; $R_{shaft}$ is the shaft resistance; $I_{b}$ is the balance current; $I_{emf}\;$is the electromotive force (EMF)-induced current; and $I_{c}$ is the shaft earthing devices (SED)-to-ground current. However, voltage differences still exist when using a passive shaft-grounding device to prevent galvanic corrosion.

According to Figure 1b, if the current on the APB can cancel the current on the rotating shaft, the voltage difference on the shaft can theoretically be close to 0 mV as in the following equations:

(2) $$I_{APB}≅\left(I_{b}+I_{emf}\right)-I_{c}$$

(3) $$V_{shaft}≅0$$

### 2.2. Design of Electrical Pitting Prevention Device

**Figure 2:**
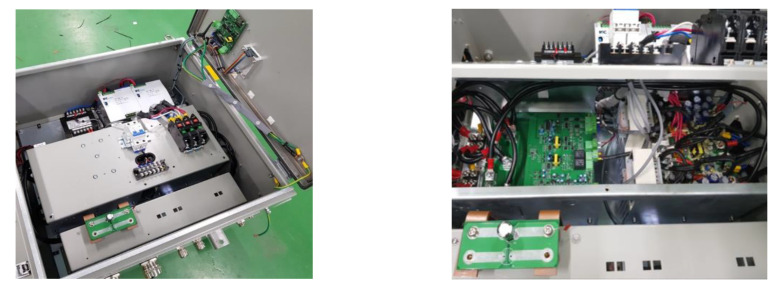
Photo of the electrical pitting prevention device.

The automatic control potential balance is the core system configuration of the electrical corrosion prevention device. It is mainly composed of a potential difference detector, power-supply control device, monitoring device, and grounding-related accessories. The potential difference detector measures the potential difference between the rotating shaft and the bearing and applies a stable and effective signal detection and noise attenuation protection circuit. It does so by using digital filters to prevent the input signal from causing damage to the system. The power supply control device mainly calculates the current control value and outputs the current to the device between the rotating shaft and the bearing. Like the potential difference sensor, it uses a noise filter and protection circuit to produce precise current output; the monitoring device is mainly a screen control for the storage of potential information including output voltage, current, measured potential difference, system operating status, and alarm. The grounding-related accessories mainly consist of a highly conductive elastic band holder and a grounding brush and holder, which transmits output current. Figure 2 is a photo of the electrical-pitting prevention device, which consists of a potential difference detector, current control value correlation device, power supply, APB informer, monitoring device, and power input and converter composed of a power control device.

**Figure 3:**
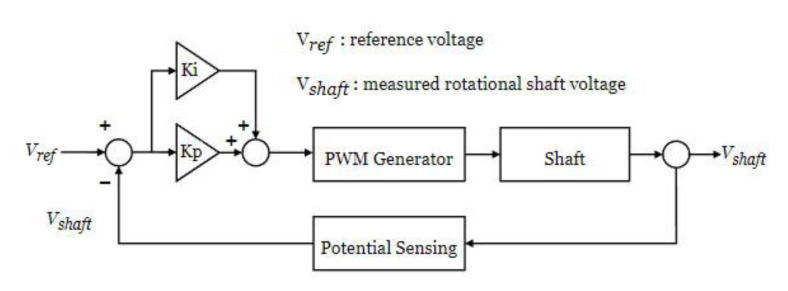
PI control algorithm.

Figure 3 depicts the system’s proportional–integral (PI) algorithm in the electrical pitting prevention device. A proportional–integral (PI) controller is a feedback control loop that calculates an error signal by taking the difference between the output of a system. Proportional gain $K_{p}$ and integral gain $K_{i}$ in the controller are 0.01 and 0.01, respectively. The PWM generated by the PI controller acts as the metal–oxide semiconductor field-effect transistor (MOSFET) gate voltage of the power controller, which generates a current that bypasses the one flowing through the rotating shaft. PWM signal was generated by the function generator (FG-281, Kenwood, Yokohama-shi of Japan), voltage was measured by the oscilloscope (MDO3024, Tektronix, Beaverton of USA) at 10 µs sampling speed, and the feedback control speed was set to produce 2 kHz frames overall. In the marine engine, reference voltage, $V_{\mathrm{ref}}$ between rotating shafts and bearings was about 20 mV. The design goal of the electrical pitting prevention device is to control the shaft voltage $V_{\mathrm{shaft}}$ to a minimum value. The PI controller continuously calculated an error value as the difference between a desired $V_{\mathrm{shaft}}$ and $V_{\mathrm{ref}}$ and eventually controlled the shaft voltage within 2 mV.

### 2.3. Experimental Setup and Test Method

**Figure 4:**
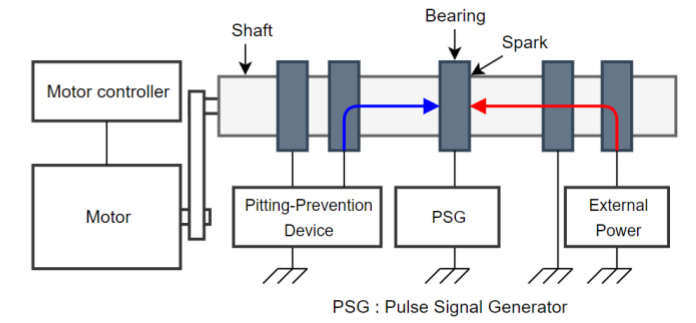
The schematic diagram of the experimental setup for electrical pitting prevention device.

**Figure 5:**
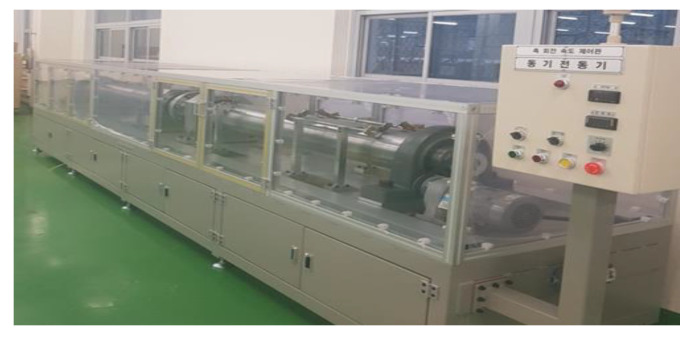
Photo of the experimental setup for electrical pitting prevention device.

To verify the operation of the rotating shaft anti-corrosion device using a power supply, an experimental bench equipped with the same motor-driven rotating shaft device as a ship or factory equipment was established. By supplying current to the ground and the rotating shaft, a potential difference between the rotating shaft and the bearing was artificially created. The motor frame was fabricated and structurally insulated so that passive- shaft grounding could be done using brushes and brush holders. The experimental bench is shown in Figure 4 and Figure 5.

The schematic diagram of the experimental setup for the electrical pitting prevention device is shown in Figure 4. The external power supply provides voltage to the rotating shaft so that it forms a potential difference with the grounded bearing. The device calculates the current corresponding to the potential difference and the flows to the rotating shaft, thereby offsetting the potential difference on the rotating shaft. The passive shaft ground (PSG) test circuit monitors the current ripple through a high-definition pulse signal.

**Figure 6:**
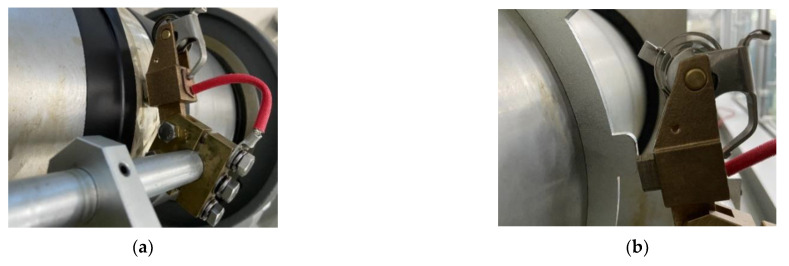
The specific coupling device: (**a**) passive shaft-grounding device and (**b**) saw-tooth-shaped connection device.

The details of the specific coupling device are shown in Figure 6. The traditional electrical carbon graphite brushes were abandoned, and conductive microfiber rings were used for shaft grounding and current conduction. This brush not only prevented voltage build-up, but also prevented current conduction at frequencies in the MHz range of tens of amps. To verify the voltage strength on the rotating shaft, a saw-tooth-shaped plate was installed as shown in Figure 6b. It was designed so that when the shaft rotated, the connection between the grounding brush and the shaft had repeated contact and separation rather than continuous contact, thereby forming a potential difference that generated sparks.

**Figure 7:**
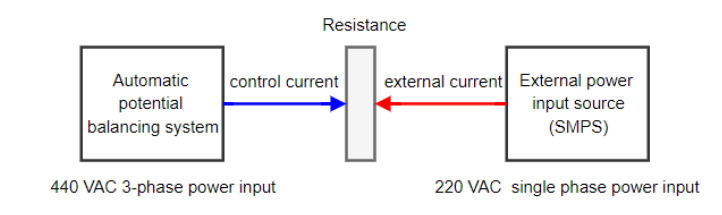
Test method for electric pitting prevention device.

In the experiment, the same rotating shaft from a ship or factory equipment was used to simulate the working state. As shown in Figure 7, the external power input source connected to the switching mode power supply (SMPS) provided a constant current of 220 V of alternating current (AC), a single-phase power input, and artificially created a potential difference. The automatic potential balancing system of the electrical pitting prevention device used a 440 V AC 3-phase power input. By calculating the voltage difference on the rotating shaft, the corresponding current automatically provided a resistance on the rotating shaft. The experiment was carried out in three steps. The first eliminated the spark generated between the grounding brush and the rotating shaft. The feasibility of the electrical corrosion protection device scheme was demonstrated by a comparison with the power supply shaft and the removal of the spark by the passive-grounding device. It is worth noting that the absence of sparks did not mean that there was no potential difference on the rotating shaft because it may have been too small to generate a spark. Therefore, further research and testing are needed to verify its performance. 

In the second step, the galvanic corrosion protection of the power supply shaft was quantified, and in the third, the electrical corrosion protection device was installed on the commercial hull shaft to further verify its performance. The goal of this study was to control the potential difference on the rotating shaft to within 2 mV. In this study, through three performance tests, an optimal design scheme for the device was proposed, and the 2 mV requirement was realized.

## 3. Experimental Results

### 3.1. Preliminary Verification of Device Performance in Laboratory

**Figure 8:**
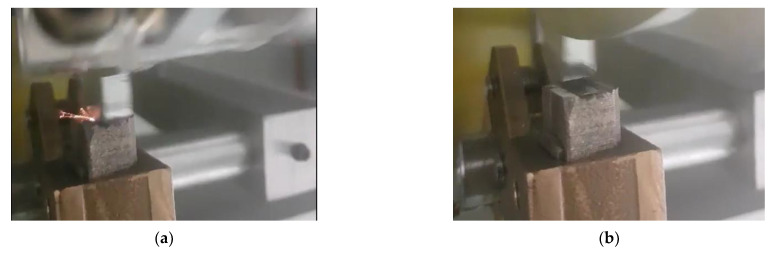
Photo of spark generation for saw-tooth-shaped plate: (**a**) passive shaft-grounding device and (**b**) electrical pitting prevention device.

To ensure the spark generation condition in the driving shaft with a passive shaft-grounding device and electrical pitting prevention device, the saw-tooth-shaped plate was specially designed and installed as shown in Figure 8. In this experiment, a constant current was supplied through an external power input source to provide the potential difference on the rotating shaft. The spark generation conditions of the saw-tooth-shaped plate were compared in two cases: when the electrical pitting prevention device was driven and when it was not. Using a high-resolution camera, the sparks generated between the plate of the rotating shaft and the grounding brush are clearly visible. As shown in Figure 8a, when the anti-corrosion device was not driven and relied only on the grounding device to prevent electrical corrosion, sparks between the saw-tooth plate and the grounding brush could clearly be seen. There was still a large potential difference, and the passive shaft grounding device could not completely prevent electrical corrosion. As shown in Figure 8b, when the anti-corrosion device was driven, there was no spark, indicating that the potential difference on the rotating shaft had been reduced to a small value. This experiment preliminarily verified that the electrical pitting prevention device had excellent performance in reducing the potential difference of the rotating shaft and preventing electrical corrosion.

**Figure 9:**
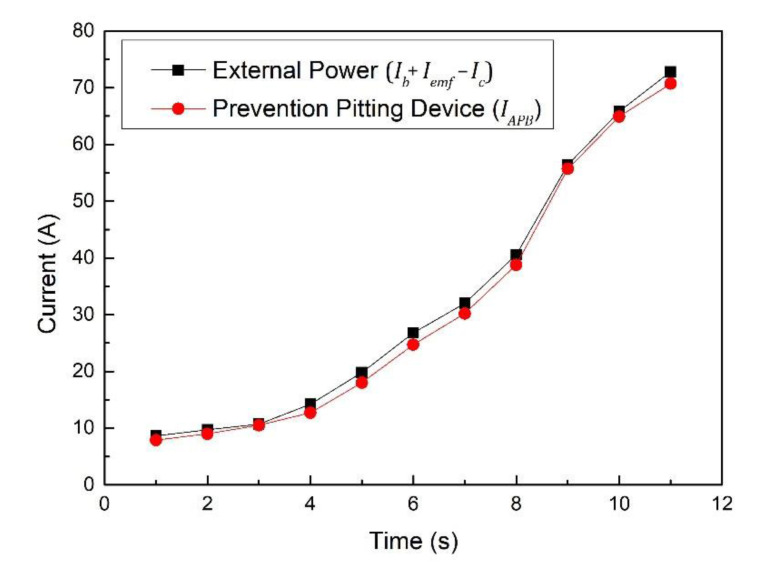
Current variation using electrical pitting prevention device to offset any external current supplied to the rotating shaft.

Figure 9 shows current variation using the electrical pitting prevention device to offset any external current supplied to the rotating shaft. The monitoring equipment fed back the output voltage and then fed the current and measured potential difference of the external power supply to the potential difference sensor and the current-control value computing device. Then, the power control device output the calculated current to the rotating shaft. The output current of the device almost canceled the output current of the external power supply. Therefore, it can be inferred that even if there was current on the rotating shaft, it must have been maintained at a very low state. To explore the specific value of the potential difference of the rotating shaft further, more refined experimental measurements are required.

### 3.2. Performance Verification for Potential Differences and Output Voltage Ripple

Table 1 shows the first test using the power shaft electrical corrosion protection device with a potential sensing voltage of 0.005 mV. The results showed that the total average potential difference was 6.12 mV, and the average ripple was 7.42 mV. The performance required for the rotary shaft galvanic corrosion prevention device using the power supply was not achieved, resulting in poor results. The potential difference was too high due to the output ripple. To keep it below 2 mV, the ripple had to be kept below 1 mV. To improve the ripple of the output power, the ripple of the input power was improved by using a smoothing circuit composed of a reactor and a capacitor at the power input. According to the potential value measured by the output/input control circuit, the change speed of the output and input currents were precisely controlled, and the sharp potential change improved.

Table 2 shows the second test using the electrical pitting protection device with a potential sensing voltage of 0.001 mV and an input-stage smoothing circuit added to reduce the ripple voltage. The results showed that the total average potential difference was 3.27 mV, and the average ripple was 3.68 mV. Although the performance test results improved from the first test, they still fell short of the performance required for a rotary shaft galvanic corrosion prevention device using a power supply. By analyzing the data, we concluded that it was impossible to remove the ripple of the potential value by only removing the ripple of the power-supply input. The measurements at the output end of the electrical corrosion prevention device also showed that the ripple of the potential value could not be removed, only improved. Therefore, the optimized solution proposed at this time is to add a smoothing circuit to the output end of the rotating shaft electrical corrosion prevention device of the power supply, which is separate from the power-supply end provided to the output unit to improve the output current ripple.

Table 3 shows the second test using the electrical pitting protection device with a potential sensing voltage of 0.001 mV and an input/output stage smoothing circuit to reduce the ripple voltage. The results showed that the total average potential difference was 0.1 mV, and the average ripple was 0.2 mV. The desired goal of reducing the potential difference to less than 2 mV was achieved, and the performance measurements were stable. Therefore, it is very important to minimize the output ripple to maximize the performance. In the working environment of the rotating shaft anti-corrosion device of the power supply, the ripple of the potential value may have increased due to the noise components generated when the power source motor and the shaft of the electric motor rotated. Therefore, when using the anti-corrosion device of the rotating shaft of the power supply, the ripple of the potential value must be kept within 1 mV to keep the potential of the rotating shaft within 2 mV.

### 3.3. Validation of Anti-Corrosion Performance of Commercial Vessel Rotating Shafts

**Figure 10:**
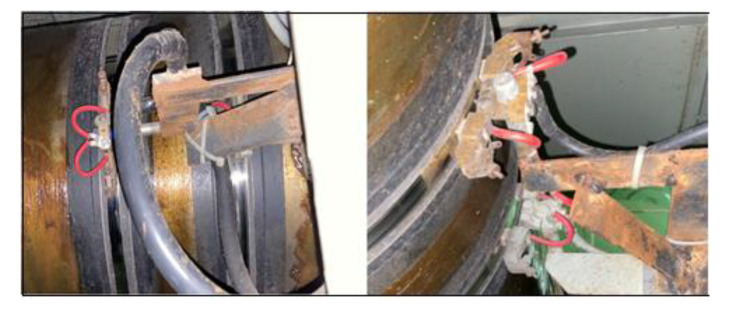
Installation of electrical pitting prevention device on the rotating shaft of the commercial vessel.

To further verify the performance of the electrical pitting protection device for the rotating shaft of a power supply in a real application, we installed the electrical pitting prevention device on the rotating shaft of a commercial vessel and detected the voltage difference under actual working conditions. The installation is shown in Figure 10.

Table 4 shows the performance verification results of applying the electrical-pitting prevention device on the rotating shaft of the actual commercial vessel. In the research, the current and potential difference on the rotating shaft were mainly recorded for a substantial amount of time. The potential difference caused by the bearing current and electrostatic field and the speed of the rotating shaft had little effect on the test results. Therefore, no value was recorded. The test log sheet had 22 valid data records, among which were the power of electrical-pitting prevention device maximum ‘resistance’current is 16.4 A and the rotating shaft maximum potential difference is 1.5 mV. The potential difference on the rotating shaft was controlled within 2 mV. Therefore, the electrical corrosion protection device of the power supply shaft reduced the potential difference on the rotating shaft and alleviated electrical corrosion.

As shown in Table 5, the shaft potential difference results show that the electrical pitting prevention device is superior in suppressing shaft current and shaft voltage compared to other solutions. The shaft potential difference was reduced to less than 2 mV, which can meet most industrial safety production requirements, and the long-term stable performance has been verified on actual commercial ships. However, the electrical pitting prevention device still has room for improvement, as the balance current is too high. As shown in Table 4, in practical applications, the balance current is kept at 11~17 A. This is not only a waste of energy, but also increases the risk of device failure. In the algorithm of the fourth arm circuit [26], the design idea of reducing the common mode voltage and thereby reducing the induced current seems to be a feasible solution to reduce the balance current.

## 4. Conclusions

In this paper, we design the electrical pitting prevention device through two design ideas of ‘dredged’ and ‘resistance’. First, the shaft current is ‘dredged’ through the passive shaft grounding device, and then the automatically controlled balance system input balance current ‘resistance’ the shaft current to balance the shaft potential difference. Finally, the shaft voltage is stably controlled within 2 mV, which can meet most industrial production safety requirements. Compared with the existing shaft electro-corrosion solution, the automatic control balance system algorithm equipped with the electrical pitting prevention device has stable performance and can be applied to equipment with a large shaft potential difference. In order to prevent the equipment from electric corrosion of the rotating shaft, a new equipment design idea is proposed.

1. By measuring the potential difference between the rotating shaft and the bearing, the current corresponding difference was calculated and sent to the rotating shaft, which eliminated the potential difference and prevented electrical corrosion.
2. To generate and remove potential differences within a few mV in rotating machines, a potential difference detector capable of measuring at least 0.005 mV was required. In addition, smooth signals had to be provided by applying noise filters and protection circuits.
3. It was necessary to apply a current-control calculation algorithm to which the PID algorithm was applied as an output current to remove the potential difference as measured by the potential difference detector as basic information.
4. A power-control device, which output the current between the rotary shaft and the bearing as calculated through the control calculation algorithm, was manufactured. Noise filters and protection circuits were applied, and the focus was on maintaining precise current output.
5. A rotating shaft test bench was established to check the performance and operational status of the manufactured parts or devices over time. It took into account various field conditions and is expected to be applied to other research areas of rotating shafts.
6. The electrical corrosion protection device can control the potential difference of the rotating shaft within 2 mV, which means that the current-control algorithm can operate normally. In addition, it was confirmed that it exhibited superior performance compared with that of the existing shaft-grounding device.

## Figures and Tables

**Table 1:** First test result with a potential sensing voltage of 0.005 mV.

No.	$V_{shaft}$			
	Min [mV]	Max [mV]	Average [mV]	Ripple [mV]
1	1.32	7.04	5.12	5.72
2	−2.23	8.05	6.54	9.82
3	0.97	7.66	5.55	5.69
4	−1.86	8.46	6.73	8.60
5	1.55	8.81	6.65	7.26

**Table 2:** Second test result using a potential sensing voltage of 0.001 mV and an input stage smoothing circuit.

No.	$V_{shaft}$			
	Min [mV]	Max [mV]	Average [mV]	Ripple [mV]
1	0.78	4.74	3.51	3.96
2	1.53	4.49	2.56	2.96
3	1.95	4.21	3.56	2.26
4	−0.12	4.89	2.95	5.01
5	0.32	4.51	3.77	4.19

**Table 3:** Third test result using a potential sensing voltage of 0.001 mV and an input/output stage smoothing circuit.

No.	$V_{shaft}$			
	Min [mV]	Max [mV]	Average [mV]	Ripple [mV]
1	0.08	0.20	0.11	0.12
2	0.01	0.18	0.09	0.17
3	0.05	0.20	0.10	0.15
4	−0.02	0.20	0.09	0.22
5	0.02	0.21	0.07	0.19

**Table 4:** The performance test log sheet of the device in a real vessel.

Date	Prevention Pitting Device		SED	Date	Prevention Pitting Device		SED
	mV	A	mV		mV	A	mV
1	0	13.2	0.6	16	0	14.8	1.2
2	0	0	0	17	0	14.6	1.5
3	0	0	0	18	0	15.1	1
4	0	12.8	0.8	19	0	15.3	0.9
5	0	11.6	0.8	20	0	16.2	1.1
6	0	12.7	0.7	21	0	16.4	0.9
7	0	13.6	0.9	22	0	15.9	1.1
8	0	12.9	1.1	23	0	15.3	0.8
9	0	14.2	1.4	24	0	0	0
10	0	0	0	25	0	14.8	0.6
11	0	0	0	26	0	13.9	0.7
12	0	13.6	1.3	27	0	13.7	0.4
13	0	0	0	28	0	13.8	0.2
14	0	11.7	1	29	0	0	0
15	0	0	0	30	0	14.1	0.6

**Table 5:** Performance comparison of anti-corrosion device.

Title 1	Passive Shaft Ground [44]	Insulating Shaft [32]	Static Charge Dissipation [37]	Pitting-Prevention Device
Shaft potential difference	100 mV	70%	4–7 V	>2 mV

## Data Availability

Not applicable.
